# SUCCESSFUL AGING IN ALASKA NATIVE ELDERS ACROSS DIVERSE REGIONS

**DOI:** 10.1093/geroni/igz038.3060

**Published:** 2019-11-08

**Authors:** Steffi M Kim, Eric Wortman, Keri Boyd

**Affiliations:** 1 University of Alaska Anchorage, Anchorage, Alaska, United States; 2 UW School of Medicine, Anchorage, Alaska, United States

## Abstract

Existing conceptualizations of successful aging are mainly based on Western cultures, ignoring the inclusion or exploration of culturally-relevant knowledge within the experience of successful aging. Lewis (2011) drew on the experiences of Elders and identified four elements of Eldership (successfully aging elders) in the Bristol Bay region of Alaska: “a) emotional well-being, b) community engagement, c) spirituality, and d) physical health” (p. 544). Given the unique and distinct environmental locations of this study, this presentation builds upon Lewis previous research and will highlight similarities and differences of Alaska Native successful aging between three rural geographic areas of Alaska. 42 Alaska Native Elders were interviewed from the Norton-Sound subregion, 21 Alaska Native Elders from the Aleutian Pribilof Islands, and 26 Elders from the Bristol Bay region. A community-based, exploratory, qualitative research methodology was used to allow for the collaboration of researchers and communities as equal partners. Qualitative interviews explored the participant’s life, influences on aging well, and their aging process. Thematic analysis was used to establish codes and main themes based on the three different cultural regions of Alaska. Results argue for the expansion and emphasize on social components, historical perspectives, and the importance of place (cultural and geographic differences), as well as generativity and gerotranscendence. Findings will be used to develop community-specific health promotion and prevention programs to help Elders find meaningful activities that promote health and teach individuals to cope with aging-related changes.

